# Successful Treatment of Histiocytic Sarcoma in Childhood

**DOI:** 10.7759/cureus.103409

**Published:** 2026-02-11

**Authors:** Haroula Tsipou, Georgia Avgerinou, Stavros Glentis, Kalliopi Stefanaki, Antonis Kattamis

**Affiliations:** 1 Division of Pediatric Hematology-Oncology, First Department of Pediatrics, National and Kapodistrian University of Athens, "Aghia Sophia" Children's Hospital, Athens, GRC; 2 Department of Pathology, National and Kapodistrian University of Athens, "Aghia Sophia" Children's Hospital, Athens, GRC

**Keywords:** chemotherapy, children, histiocytic sarcoma, immunochemistry, langerhans cell histiocytosis (lch)

## Abstract

Histiocytic sarcoma (HS) is a rare malignant neoplasm that belongs to malignant histiocytoses. HS primarily occurs in adult males and is exceptionally uncommon in pediatric populations. Extranodal regions represent the most common sites of HS manifestation. Diagnosis necessitates histopathological evidence of neoplasm, along with verification of cellular origin through specialized studies. Here, we describe a case of a three-year-old female toddler with HS in her calf that was successfully treated with the chemotherapy protocol of Langerhans Cell Histiocytosis (LCH)-IV, the International Collaborative Treatment Protocol for Children and Adolescents with LCH, version 1.3.

## Introduction

Histiocytic sarcoma (HS) is a rare malignant neoplasm that, according to the 5^th^ edition of the World Health Organization (WHO) Classification of Haematolymphoid Tumours, is classified in histiocytic neoplasms [1]. This group of tumors is derived from common myeloid progenitors that give rise to cells of the monocytic/histiocytic/dendritic lineage. The overall incidence of HS is reported to be below 0.17 per 1,000,000 individuals. HS primarily develops in adults of all ages, although its incidence peaks during the sixth to seventh decades of life. There is a slight male predominance, and only a limited number of pediatric cases have been documented [2]. The etiology of HS is uncertain. Extranodal areas such as the respiratory system, gut, lung, and nasal cavities are the most frequent sites of development of HS. The clinical manifestations of HS vary from localized to broadly disseminated disease [3]. Because of its rarity, there are no standard therapies for patients with HS. Patients presenting with disease involving critical organs such as the central nervous system or liver pose significant management challenges. Analysis of the National Cancer Database (2004-2015) revealed that systemic chemotherapy alone was administered to 25% of patients, while surgery alone was performed in 22%, and radiotherapy alone in 4%. Hematopoietic stem cell transplantation was utilized in approximately 3% of cases [4]. Surgical intervention is regarded as the preferred treatment modality for unifocal or localized disease. The progression to unresectable, disseminated, or metastatic HS is linked to an unfavorable prognosis [5]. In the past two decades, a deeper understanding of the molecular mechanisms that drive HS has resulted in new treatment modalities targeting MEK and BRAF. Owing to the high expression of programmed death ligand 1 (PD-L1), few case reports have described treatment with PD-L1 inhibitors [6]. Currently, there are no large-scale randomized clinical trials available for HS. Here, we described a case of a three-year-old female toddler with HS in her right lower extremity.

## Case presentation

**Figure 1:**
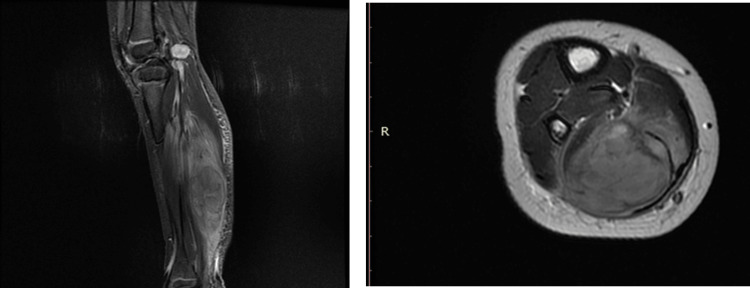
MRI with contrast of the diagnosis (coronal, axial)

**Figure 2:**
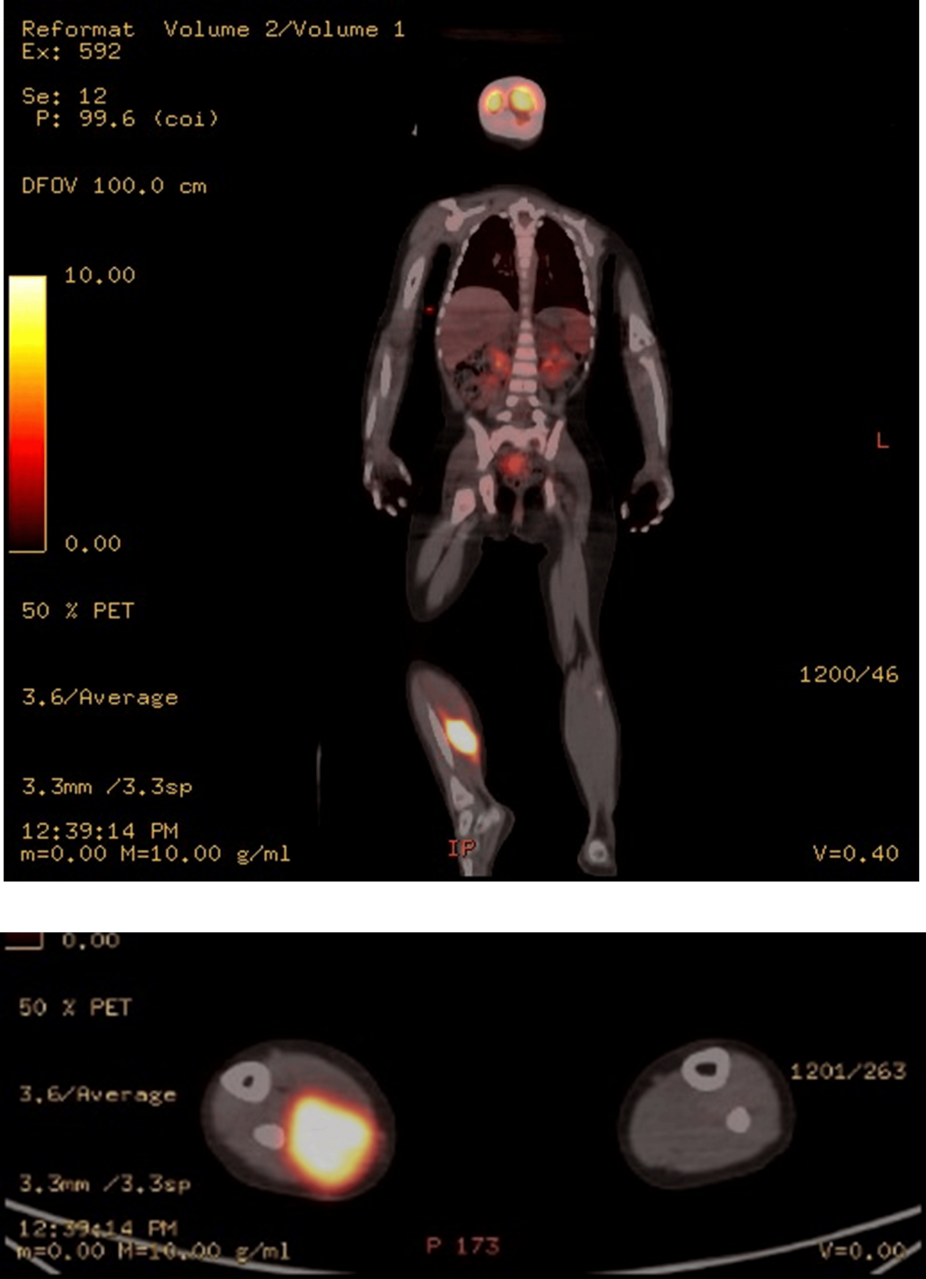
PET-CT of the diagnosis (whole body and axial of thighs)

A three-year-old female toddler was presented with pain and limpness of her right lower extremity. Upon clinical examination, she had a palpable mass on her right calf. MRI of the right lower extremity showed a mass (diameter 5.5 cm x 2.9 cm x 3.1 cm) with two adjacent smaller lesions (diameter 0.9 x 0.8 x 0.9-1.2 cm x 0.9 cm x 1.3 cm) at the calf. The 18-fluorodeoxyglucose-positron emission tomography-computed tomography (18FDG-PET/CT) revealed an increased uptake on the right calf (maximum standardized uptake value (SUVmax) 12.8) and on the right groin (SUVmax 1.7) (Figures 1, 2).

The CT of the thorax and the bone scan were negative for metastases, and the bone marrow aspiration and trephine did not show bone marrow involvement.

The histological examination showed a malignant oxyphilic epithelioid neoplasm composed of a diffuse population of medium-sized and large neoplastic cells with well-delineated eosinophilic cytoplasm, a nucleus with fine chromatin, cerebriform with a focal, obvious nucleolus, moderate/severe nuclear atypia, mild/moderate mitotic activity (up to one to four mitoses), and the presence of atypical mitoses.

**Figure 3:**
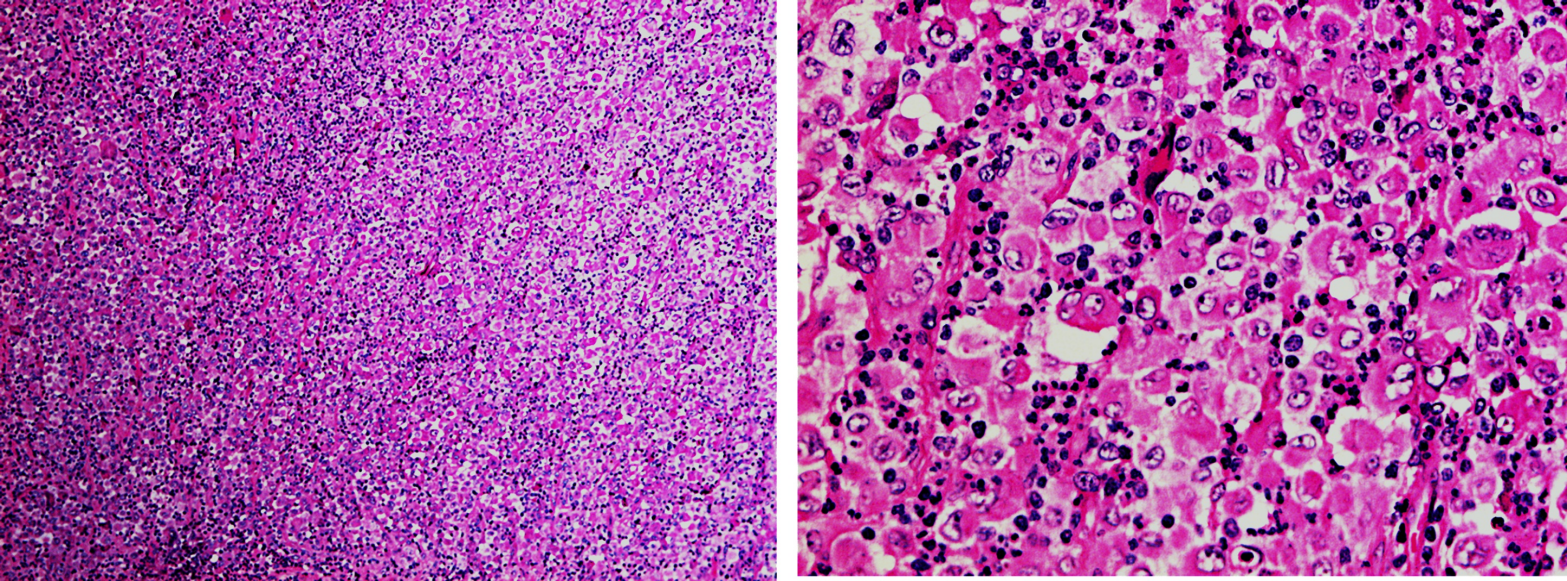
H&E biopsy of the tumor

Characteristic features were a) the presence of giant mononuclear cells with a round nucleolus or an embryo-like nucleus and the presence of rare binuclear neoplastic cells with an obvious nucleolus; b) the presence of multinuclear cells with a horseshoe or multilobated nucleus; c) the presence of apoptotic necrosis (~20%); d) the considerable population of small lymphocytes, plasma cells, a lot of polymorphs, and eosinophils with suppuration; e) focal hemophagocytosis (Figure 3).

The immunohistochemistry of the tumor showed diffuse expression of CD68/PG-M1 (diffuse dot cytoplasmic), CD163 (membranous and cytoplasmic), fascin (cytoplasmic), S-100 protein (~20-25%), HLA-DR (>40% mostly membranous), CD4 (heterogenous), focal expression of factor ΧΙΙΙa (~5%), and CD45RO (<5%). 

There was no expression of CD30/BerH2, ALK-1/p80, granzyme B, EMA, CD1a, langerin, CD21, CD23, CD21, CD23, CD20/L-26, PAX-5/BSAP, CD5, CD7, CD2, CD8, CD43, myeloperoxidase, CD15, CD33, CD34, CD13 (weak focal expression in~10%), c-kit, CD61, CD71 (few neoplastic cells+), CD56/NCAM, cytokeratin 8.18, desmin, smooth muscle actin, and HMB45.

**Figure 4:**
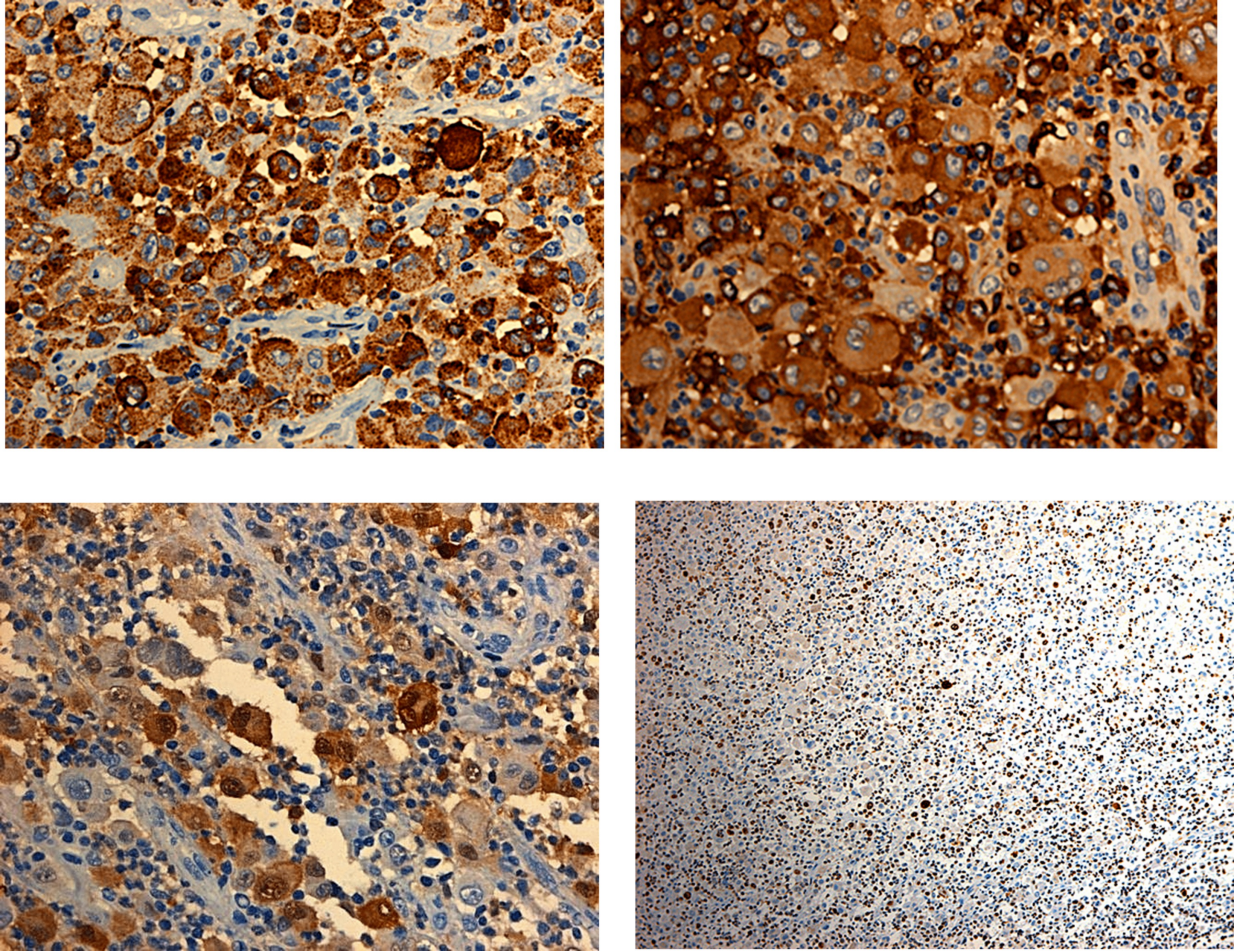
Immunohistochemistry of the tumor biopsy (CD168, CD163, S-100 protein, Ki-67/MIB-1)

The protein product of the INI-1/SMARCB1 gene was retained in >99% of the nuclei of the neoplastic cells, the Ki-67/MIB-1 proliferative index was detected in 20% to 25% of the nuclei, and S-100 was positive (Figure 4).

The morphological and immunophenotypic features of the tumor were consistent with a histiocytic hyperplastic lesion (HS).

Reverse transcription-polymerase chain reaction (RT-PCR) at tumor tissue was negative for BRAF V600E, V600K, V600D, V600R, and MAP2K1 (EXONS 2&3). RT-PCR for the N-terminus of nucleolar phosphoprotein-anaplastic lymphoma kinase (NPM-ALK) and T cell receptors β (TCRβ), TCRγ, and TCRδ genes in the blood were also negative.

Our patient has a male cousin who was diagnosed with mixed-phenotype acute leukemia (MPAL) at the age of five years old. With this familiar history, we proceed to targeted next-generation sequencing of our patient, her parents, and her cousin, with no pathological findings.

The patient was treated with systemic chemotherapy according to Langerhans Cell Histiocytosis (LCH)-IV, the International Collaborative Treatment Protocol for Children and Adolescents with LCH, version 1.3 [7]. The patient received initial chemotherapy with two blocks of cytosine arabinoside (ARA-C)/2-chlorodeoxyadenosine (2-CDA) (STRATUM III) [8].

After the first block, a partial response was documented.

After the second block MRI of the lower right extremity showed only abnormal contrast enhancement without the presence of a tumor, the response was categorized as Active Disease (AD) Better, and the patient continued treatment with continuation therapy (Part 1: two courses of 2-CdA; Part 2: 12 pulses of vinblastine (VBL)/prednisone (PRED), mercaptopurine (6-MP), and methotrexate (MTX) for 24 weeks; Part 3: 6-MP, MTX for 12 months), as per protocol.

**Figure 5:**
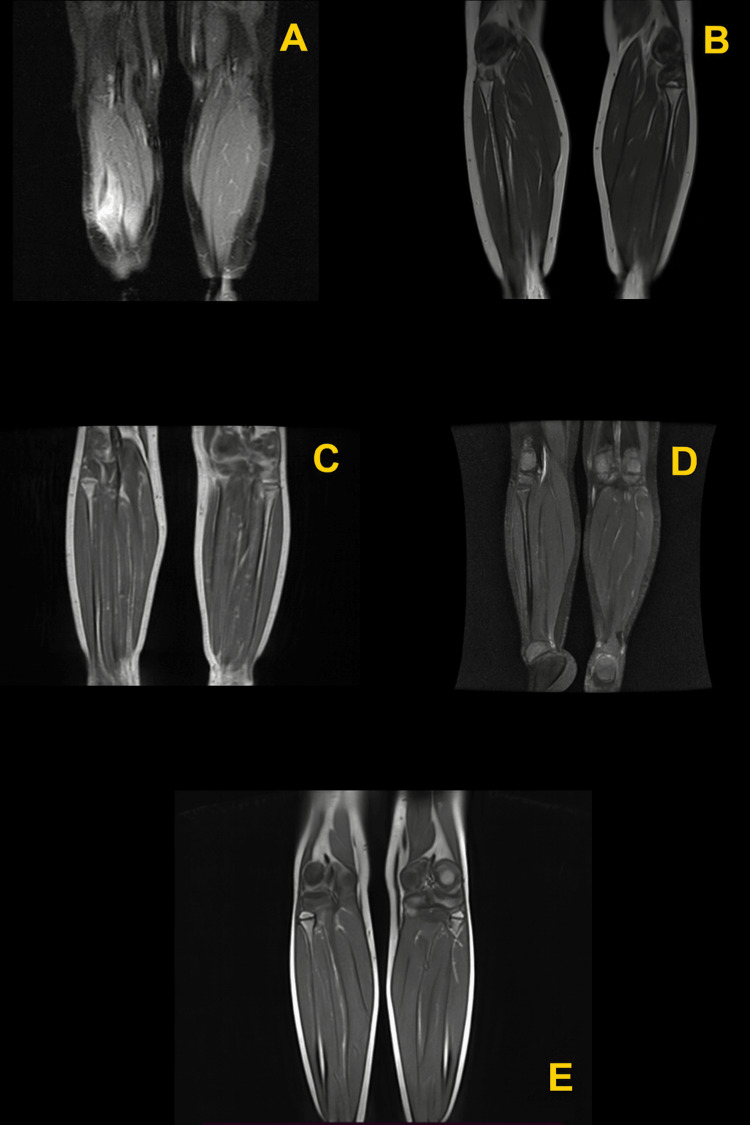
MRI with contrast of the lower extremities A. after initial chemotherapy; B. after continuation therapy part 1; C. after continuation therapy part 2; D. six months after end of therapy; E. 2.5 years after end of therapy

All subsequent MRI images showed no active disease (Figure 5). As of the last evaluation, the patient has remained in remission for 6.5 years post end of therapy.

## Discussion

HS is a very rare hematopoietic neoplasm from non-Langerhans histiocytic cells, mostly described in male adults with a wide age distribution (median age 63 years old). Its incidence is 0.17 per 1 000 000 individuals [9]. The occurrence at such a young age, like our patient, is very rare, as only limited case reports in the pediatric population have been published [10-12]. The most frequent sites for HS are extranodal (gastrointestinal tract, superficial and deep soft tissue, lung, and nasal cavity), but there have also been described cases of HS of the brain, skin, and lymph nodes [3]. It may be presented either as a localized tumor, like our case report, or as a disseminated disease. There have been described patients with HS with a history of other hematological neoplasias like lymphomas, chronic lymphocytic leukemia, and multiple myeloma [3]. In pediatric patients with HS, association with autoimmune lymphoproliferative syndrome, acute leukemia, and following CAR-T cell therapy has been reported [11,12].

The histological diagnosis of HS is challenging, as it overlaps with many other entities, like lymphomas, melanomas, sarcomas, and histiocytic/dendritic cell tumors [3]. The immunochemistry is helpful in the establishment of a diagnosis. Most HS express CD68, CD163, and PU.1, with a subset of cases also expressing CD31, CD4 (cytoplasmic), and CD45RO. Expression of at least two of the following markers (CD68, CD163, CD4, and lysozyme) has been recommended as a diagnostic criterion in HS [13]. The expression of CD163 has been proven to be a very significant marker in the diagnosis of HS [14]. Another significant criterion for diagnosis is the no expression of Langerhans cell (CD1a, langerin), follicular dendritic cell (CD21, CD35), myeloid cell (CD13, MPO), melanocytic (SOX10, HMB-45, MART-1), epithelial (keratin, EMA), vascular (ERG), and specific B-cell and T-cell markers (CD20, PAX5, CD3).

There is no recommended specific therapy for the HS. The therapeutic options in children with HS are surgical excision of localized tumors, radiotherapy, and/or chemotherapy. Chemotherapy, which is usually used, is based on lymphoma protocols like cyclophosphamide, doxorubicin, vincristine, and prednisone (CHOP); ifosfamide, carboplatin, and etoposide (ICE); doxorubicin, bleomycin, vinblastine, and dacarbazine (ABVD); or LCH protocols [15-18].

Recent reports focused on the molecular profiling of the HS, which may guide relevant targeted therapy [18,19]. The most frequent mutations involved the RAS-MAPK pathway (MAP2K1, KRAS, NRAS, BRAF, PTPN11, NF1, CBL), and in some cases, mutations in the PI3K signaling pathway (PTEN, MTOR, PIK3R1, PIK3CA) [20]. Recent research has indicated a significant prevalence of PD-L1 expression, identifying it as a promising target for therapeutic intervention [21].

## Conclusions

HS is a hematopoietic neoplasm originating from non-Langerhans histiocytic cells and is exceedingly rare in pediatric patients. Histological diagnosis of HS poses significant challenges due to its overlap with various other entities. The diagnostic criteria that have been suggested through the bibliography are the expression of a minimum of two of CD68, CD163, CD4, and lysozyme, with CD163 being the most significant in diagnosis. There are currently no specific chemotherapy protocols established for children. The therapeutic options mostly presented in case reports are surgery, radiotherapy, or a combination of them. Notably, despite the rarity of both the patient’s young age and the atypical presentation as a soft tissue tumor, our case demonstrated an excellent response to the standard LCH protocol. The future directions for such rare and aggressive sarcomas are the establishment of well-designed clinical trials, which would provide evidence-based chemotherapy protocols, including immunotherapy and targeted therapy.
